# Combined pretreatment with ^18^F-FDG PET/CT and Comet assay guides the concurrent chemoradiotherapy of locally advanced cervical cancer: study protocol for a randomized controlled trial

**DOI:** 10.1186/s13063-018-2800-7

**Published:** 2018-08-03

**Authors:** Shengjun Ji, Qunchao Hu, Jiahao Zhu, Jie Chen, Qingqing Chen, Zhengcao Liu, Chen Shen, Ru Yang, Haoyao Sun, Jinchang Wu, Ke Gu

**Affiliations:** 1 Department of Radiation Oncology, The Affiliated Suzhou Hospital of Nanjing Medical University, 16 Bai Ta Road, Suzhou, Jiangsu 215001 People’s Republic of China; 2 Department of Clinical Oncology, Nanjing Medical University, Nanjing, Jiangsu China; 3 Department of Gynecology, The Affiliated Suzhou Hospital of Nanjing Medical University, Suzhou, Jiangsu China

**Keywords:** Locally advanced cervical cancer, Radiosensitivity, PET/CT, Comet assay

## Abstract

**Background:**

Cisplatin-based chemoradiation is the standard of care for patients with locally advanced cervical cancer. Nevertheless, an increasing number of radio-resistant tumors still recur.

**Methods and design:**

Three hundred cervical cancer patients with FIGO stages IB2–IVA and no para-aortic lymphadenopathy (> 10 mm) will be enrolled. All patients will be randomly divided into four arms to receive either (1) intensity modulated radiation therapy (IMRT), (2) RapidArc, (3) positron emission tomography/computed tomography (PET/CT) with F-18 fluorodeoxyglucose (FDG), or (4) Comet assay-guided IMRT, PET/CT, and Comet assay-guided RapidArc. All patients will receive definitive radiotherapy consisting of external beam whole pelvic radiation therapy and high-dose rate intracavitary brachytherapy. Cisplatin 30 mg/m^2^ weekly will be administered concurrently for five courses. Two to four cycles of TP (Taxol 135 mg/m^2^, D1, and DDP 75 mg/m^2^, D1–3) sequential chemotherapy will be performed according to MRI or PET/CT after cisplatin-based chemoradiation. The primary outcome measure is progression-free survival, and the second outcome measures are overall survival and time to progression.

**Discussion:**

RapidArc has an obvious advantage in improving the degree of target coverage, improving organs at risk, sparing healthy tissue, and significantly reducing the treatment time. FDG-PET/CT can increase the agreement between biopsies and delineated tumor volume and has the potential to positively impact the course of treatment. The Comet assay is attractive as a potential clinical test of tumor radiosensitivity. During radiotherapy, accurately defining disease areas is critical to avoid the unnecessary irradiation of normal tissue. Based on FDG-PET/CT and Comet assay, higher doses can be safely delivered to accurate tumor volumes, while the doses to the bladder and rectum are relatively low.

**Trial registration:**

ClinicalTrials.gov Protocol Registration and Results System Receipt Release Date: May 21, 2017 – Retrospectively registered. NCT03163979.

## Background

Cervical cancer is the most common gynecological malignancy and the second leading cause of cancer mortality in women. Nearly 500,000 new cases of cervical cancer are reported worldwide each year [1]. Although cervical cancer is often curable if detected early, a significant number of patients present with locally advanced cervical cancer (LACC) at the time of diagnosis [2]. Cisplatin-based chemoradiation (CCRT) has been considered the standard of care for patients with LACC. However, patients with stage III and IVA tumors have 5-year survival rates of only 40% and 15%, respectively [2]. Moreover, gastrointestinal and genitourinary toxicity is a concern. Grade III radiation cystitis and proctitis rates are in the range of 3–15% after radiation alone. In combination with chemotherapy, the toxicity of the treatment may be even higher [3, 4]. Future advancements in the treatment of LACC might rely on more effective and better-tolerated therapies. Therefore, it is urgent to develop novel therapeutic approaches to treat cervical cancer.

RapidArc is a type of volumetric-modulated arc therapy. It uses progressive variations of the instantaneous dose rate, multileaf collimator positions, and gantry rotation speed to optimize dose distribution. It has potential dosimetric benefits and reduces the treatment delivery time while maintaining equivalent plan quality for prostate cases. Cozzi et al. [5] concluded that, compared with forward-planned intensity modulated radiation therapy (f-IMRT), a RapidArc plan for cancer of the cervix uteri improved the sparing of organs at risk (OARs) with uncompromised target coverage.

Patients with LACC are treated with the same pattern of CCRT, showing improved local control and survival. Nevertheless, an increasing number of radio- and chemo-resistant tumors still recur [6, 7]. This may be related to the individual biological heterogeneity and the intrinsic radiation sensitivity of cervical cancer cells. If mechanisms to improve tumor cell treatment sensitivity could be identified and/or if the tumor response could be predicted, it should be possible to improve local control and survival. Some studies have indicated that positron emission tomography/computed tomography (PET/CT) with F-18 fluorodeoxyglucose (FDG) and Comet assay have important clinical value in predicting the prognosis and radiation sensitivity of cancer treatment, which may be helpful for individualized CCRT [8–12].

FDG-PET/CT has good sensitivity for detecting the sites of disease in cervical cancer. Some studies have indicated that FDG-PET/CT can be used to measure the standard uptake value of each region and cell proliferation activity in the tumor to develop a more accurate radiation treatment plan to improve the prognosis of the patients’ disease [12–15]. The maximum standard uptake value (SUVmax) of PET/CT has been demonstrated to predict the local tumor response and survival [16–18]. The NCCN guidelines recommend that baseline PET/CT should be evaluated before CCRT is performed in LACC patients.

The Comet assay, also known as single-cell gel electrophoresis analysis, can evaluate the sensitivity of the tumor to radiotherapy, the proportion of active and hypoxic tumor cells, and the heterogeneity of the tumor response to treatment [9, 19–22]. Preclinical studies have shown that the Comet assay is consistent with the classic clone formation assay, which is a potential method for the clinical detection and analysis of radiation sensitivity [20, 23]. Using the Comet assay, higher doses of irradiation can be delivered to the tumor or a tumor region with low sensitivity to achieve individualized radiotherapy.

Based on this background, we conducted a phase II multi-institutional clinical trial. The hypothesis of the current study is that CCRT and sequential chemotherapy are safe. Comet and FDG-PET/CT-guided RapidArc-IMRT will improve survival in terms of time to progression, enhance progression-free survival (PFS) and overall survival, and lead to less treatment-related toxicity.

## Methods and design

### Trial organization

This trial was designed by the Department of Radiation Oncology and Integrative Oncology in cooperation with the Department of Interventional Radiology. The trial is an investigator-initiated trial and is supported partly by Jiangsu Province Key R&D Projects BE2015645 and the Suzhou Cancer Clinical Medical Center Szzx201506.

### Study design

**Fig. 1:**
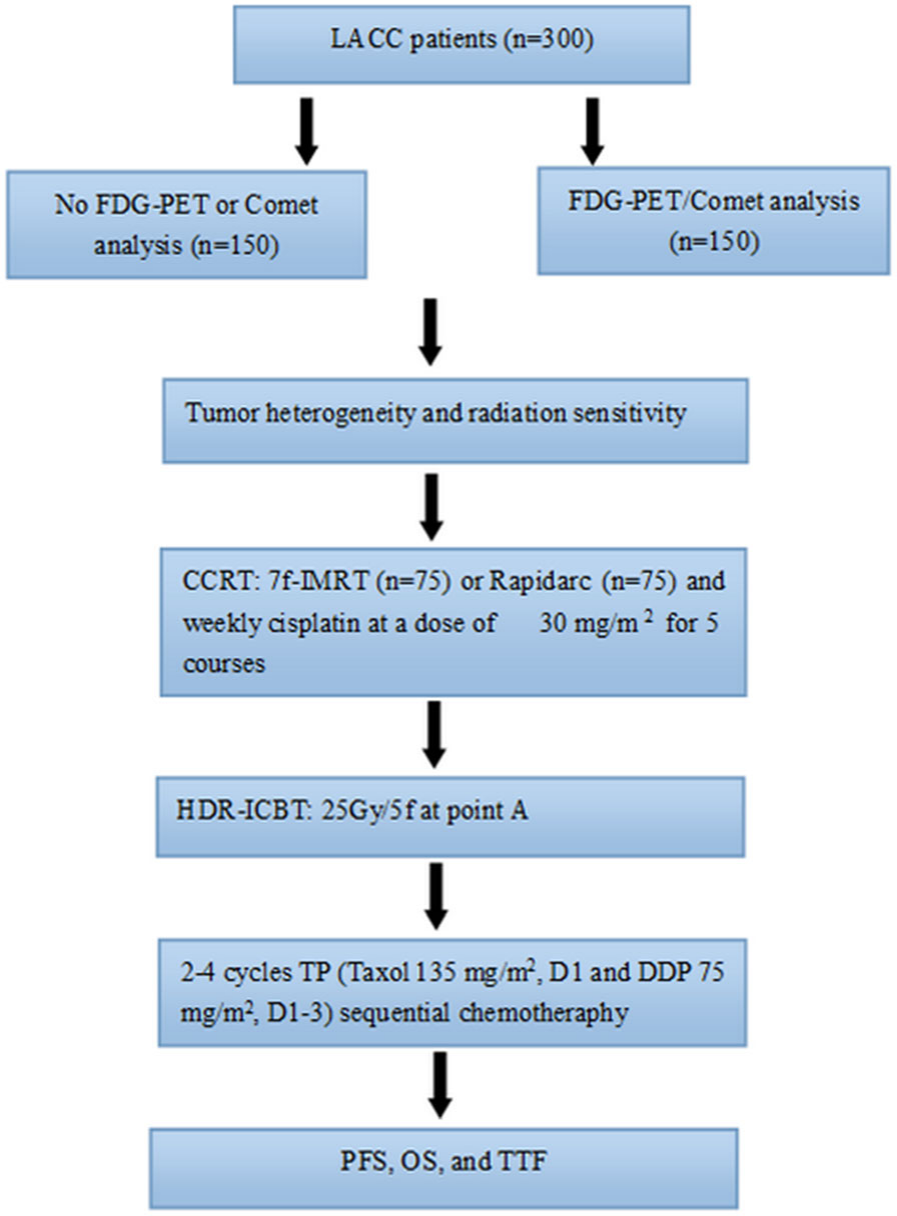
Study design

**Fig. 2:**
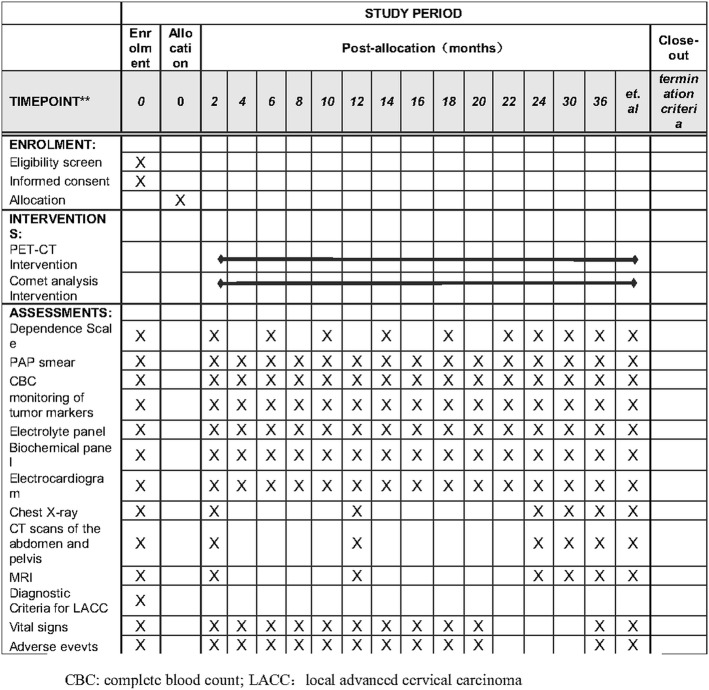
Study process schedule (according to the Standard Protocol Items: Recommendations for Interventional Trials (SPIRIT) guidelines)

The present study is a prospective, randomized, multicenter phase II/III clinical trial. The treatment schedule is shown in Fig. 1. The non-pharmacological interventions and the Standard Protocol Items: Recommendations for Interventional Trials (SPIRIT) Checklist for the implementation of study protocols were followed (Fig. 2). The dose-limiting toxicity has been defined, and safety and tolerability will be evaluated weekly during the study period. Toxicity is graded according to the toxicity criteria of the Radiation Therapy Oncology Group (RTOG) and the European Organization for Research and Treatment of Cancer (EORTC) [24]. Dose-limiting toxicity is defined as (1) grade 3 perineal skin reaction (Table 1); (2) grade 3 and grade 4 genitourinary reaction toxicity (Table 1); (3) grade 3 and grade 4 toxicity in the lower gastrointestinal tract, including the pelvis (Table 1); and (4) grade 3 and grade 4 hematological toxicity (Table 2).

**Table 1:** RTOG acute reaction grading

	Grade 1	Grade 2	Grade 3	Grade 4
Skin	Follicular, faint or dull erythema / epilation / dry desquamation / decreased sweating	Tender or bright erythema, patchy moist desquamation /moderate edema	Confluent,moist desquamation other than skin folds, pitting edema	Ulceration, hemorrhage,necrosis
Genitourinary	Frequency of urination or nocturia is twice as much as before / dysuria, urgency not requiring medication	Frequency of urination or nocturia that is less frequent than every hour. Dysuria, urgency, bladder spasm requiring local anesthetic (e.g., Pyridium)	Frequency with urgency and nocturia hourly or morefrequently/dysuria, pelvis pain or bladder spasm requiring regular, frequent narcotic/gross hematuria with/ without clot passage	Hematuria requiring transfusion/acute bladder obstruction not secondary to clot passage, ulceration, or necrosis
Lower G.I. including pelvis	Increased frequency or change in quality of bowel habits not requiring medication / rectal discomfort not requiring analgesics	Diarrhea requiring parasympatholytic drugs/ mucous discharge not necessitating sanitary pads/rectal or abdominal pain requiring analgesics	Diarrhea requiring parenteral support/severe mucous or blood discharge necessitating sanitary pads/abdominal distention	Acute or subacute obstruction, fistula or perforation; GI bleeding requiring transfusion; abdominal pain or tenesmus requiring tube decompression or bowel diversion

**Table 2:** Radiation Therapy Oncology Group (RTOG) acute hematological reaction grading

	Grade 1	Grade 2	Grade 3	Grade 4
WBC (X 1000)	3.0 to < 4.0	2.0 to < 3.0	1.0 to < 2.0	< 1.0
Platelets (X 1000)	75 to 100	50 to < 75	25 to < 50	< 25 or spontaneous
Neutrophils (X 1000)	1.5 to 1.9	1.0 to < 1.5	0.5 to < 1.0	< 0.5 or sepsis
Hemoglobin (GM %)	11 to 9.5	< 9.5 to 7.5	< 7.5 to 5.0	< 5.0

### Study objectives

The study is designed to investigate the safety, tolerability, and efficacy of radiation sensitivity-guided CCRT for LACC patients. Clarifying the safety and tolerability of two types of CCRT (RapidArc-IMRT vs. IMRT) during treatment is the primary outcome measure. Based on FDG-PET/CT and the Comet assay, higher doses of radiation can be delivered to low sensitivity tumor regions to achieve individualized treatment. The primary outcome measure is PFS, with secondary outcome measures being overall survival and time to progression.

### Coordination and monitoring

The trial is being coordinated by the clinical trial office of Nanjing Medical University, Suzhou, China. During patient recruitment in this center, on-site monitoring is performed according to good clinical practice guidelines. The trial is registered with ClinicalTrials.gov (NCT03163979). Data management is performed by the clinical trial office of Nanjing Medical University, Suzhou, China.

### Ethical approval

The final protocol was approved on March 11, 2015, by the Research Ethics Committee of Nanjing Medical University (Reference number: K20150001).

### Study sample size and grouping method

A total of 300 patients will be enrolled in the study. Due to the sample size, a block randomization procedure with randomly chosen block sizes is being used to assign participants to each group (1:1 ratio), resulting in one intervention and one control group. This method helps to maintain the balance of treatment assignment while reducing the potential for selection bias. Randomization is performed with random number generator of SPSS 20.0 software.

### Patient recruitment

All inclusion criteria should be satisfied, as follows:

1. Age: 18–70 years old;
2. Histology- or cytology-confirmed cervical squamous cell carcinomas;
3. 2009 FIGO stage including IB2, IIA2, IIB–IVA;
4. Performance status: 0–1;
5. Peripheral blood meets the following conditions: neutrophil count > 2.0 × 10^9^/L, white blood cell count > 4.0 × 10^9^/L, platelet count > 100.0 × 10^9^/L;
6. Liver and kidney function meet the following conditions: bilirubin < 1.5 mg/dL, AST and ALT more than twice the upper limit of normal serum creatinine < 1.5 mg/dL, creatinine clearance > 50 mL/min, ALP < 5 × UNL;
7. Coagulation function: International Normalized Ratio <  1.5 times the upper limit of normal ratio, and prothrombin time or activated partial thromboplastin time <  1.5 times the upper limit of normal ratio;
8. Signed informed consent before treatment;
9. Women of childbearing potential must have a negative urine or serum pregnancy test within 24 h prior to receiving the first dose of study medication;
10. Females should not be breastfeeding (even if this is not possible because of the women’s health status).

Exclusion criteria:

1. There is no definite pathological diagnosis;
2. Clinical or imaging examination reveals distant metastases;
3. Pelvis has received radiotherapy;
4. Patients cannot participate in the study because of the association with other diseases;
5. Patients cannot sign the informed consent form because of mental disorders;
6. Uncontrolled active infection;
7. No follow-up;
8. A woman of childbearing potential who has a positive urine pregnancy test within 72 h prior to allocation;
9. Subject is pregnant or breastfeeding, or expecting to conceive within the projected duration of the study, starting with the screening visit through 120 days after the last dose of trial treatment;
10. Subject has a history or current evidence of any condition, therapy, or laboratory abnormality that might confound the results of the study, interfere with the subject’s participation for the full duration of the study, or is not in the best interest of the subject to participate, in the opinion of the treating physician;
11. Subject is currently participating and receiving study therapy from another clinical trial;
12. Subject had participated in a study of an investigational agent or has used an investigational device within 4 weeks prior to the first dose of study treatment;
13. Patient who is not willing to sign the consent form;
14. Legal incapacity or limited legal capacity patients receiving other oncology-specific medication not authorized in the protocol;
15. Has had prior chemotherapy, targeted small molecule therapy, or radiation therapy prior to Cycle 1 Day 1.

Termination criteria:

1. Treatment-related serious adverse events or serious adverse reactions;
2. Patients ask to be withdrawn from the study;
3. Disease progression during treatment;
4. A violation of the research plan;
5. Patient death;
6. The researchers determine that the patient should end the clinical trial.

### Interventions

#### PET/CT and Comet assay before radiation therapy

All patients with cervical cancer treated definitively with IMRT in this study underwent an FDG-PET/CT simulation to aid in target volume delineation. The cervical cancer and lymph node metastasis was defined, and a tumor region of SUVmax > 5 was obtained. Tumor tissue samples were obtained, and the Comet assay was used to detect the initial DNA damage to understand the heterogeneity of the tumor and individual differences. Tumor cells were given 5 Gy irradiation and, once again, the Comet assay was performed to understand the radiation sensitivity of the tissue specimens.

Based on FDG-PET/CT and the Comet assay, the tumor region for which the SUVmax was higher than 5 and/or the Comet assay suggested lower radiation sensitivity was delivered a 55 Gy dose.

### Radiation therapy

#### Simulation, target, and OAR delineation

Before radiation, all patients were immobilized with a vacuum bag and received CT simulation in the supine position using a 4D CT scan (GE, USA). The slice thickness was 5 mm, and the scanning range was from the L2 vertebral body to 5 cm below the ischial tuberosities.

All structures, including the gross target volume (GTV), clinical target volume (CTV), plan target volume (PTV), and all OARs, were contoured by the radiation oncologists. As no consensus guidelines for the definitive treatment of cervical cancer exist, we used the protocol proposed by Lim et al. [25] and Forrest et al. [26]. GTV covered the cervix and any vaginal or uterine involvement, including the tumor region with a SUVmax > 5 and/or where the Comet assay suggested that the radiation sensitivity was low. The CTV covered the entire cervix and uterus, parametria, uterosacral ligaments, presacral nodes, and common iliacs. The involved lymph nodes were marked as ‘GTVnd’ and were expanded 5 mm uniformly. CTV was added to a 7-mm margin to create the PTV.

The OARs included the bladder, rectum, small bowel, and pelvic bones. The contouring of the small intestine started 2 cm above the PTV in a cranial-cordial direction and included a volume surrounding the loops of the small bowel out to the edge of the peritoneum. The rectum included from the anus to the point where it joined the sigmoid colon.

#### Intensity-modulated radiotherapy (IMRT)

Conventional fractionation was used in all patients for a total dose of 45–50.4 Gy with 6 MV high-energy photons. Based on FDG-PET/CT and the Comet assay, the tumor region with SUVmax > 5 and/or the region where the Comet assay suggested the radiation sensitivity was low was delivered a 55 Gy dose. All plans were generated using a Varian Eclipse v8.6 treatment planning system. Dose calculation followed an anisotropic analytical algorithm. Dose distribution was calculated with clinically acceptable accuracy. The f-IMRT gantry angles were 0°, 51°, 102°, 153°, 204°, 255°, and 306°, with 20 intensity levels and a dose rate of 400 monitor units min^– 1^. Doses were delivered using the step-and-shoot method.

For the rectum, the maximum dose limit was 47.5 Gy in combination with two complementary constraints: D35% < 40 Gy and D50% < 30 Gy (Dx% is the dose received by x% of the volume). Similarly, for the bladder, the maximum dose was set at 47.5 Gy in combination with D40% < 40 Gy and D50% < 35 Gy. For the region containing the small bowel, the maximum dose was limited to 47.5 Gy, while complementary constraints were specified as D30% < 30 Gy and D50% <  25 Gy.

#### RapidArc

A maximum DR of 600 monitor units min^– 1^ was set to compare the seven f-IMRT angle treatment times. Two 360° coplanar arcs (one clockwise arc rotated from 181° to 179° and the other counter-clockwise arc rotated from 179° to 181°) sharing the same isocenter were used. Collimator rotations of the first and second arc were set at X° and (360-X)° to minimize the contribution of the tongue-and-groove effect to the dose. The final dose was calculated using the anisotropic analytical algorithm (AAA_10028) accounting for inhomogeneous tissue.

#### High-dose rate intracavitary brachytherapy (HDR-ICBT)

HDR-ICBT was performed twice a week with a fraction dose of 5 Gy prescribed at point A using Ir^192^. The total HDR-ICBT dose was 25 Gy at point A. The total dose was the summation of the external beam radiation therapy doses, and HDR-ICBT doses ranged from 70 to 75 Gy. Source dwell patterns were determined according to the Manchester system [27]. A dose calculation was performed for each application using two orthogonal radiographs. The isodose curves were plotted and doses at the rectum and bladder were calculated according to the 38 criteria of the International Commission on Radiation Units and Measurements [28].

### Treatment adjustment of radiotherapy with severe toxicity and side effects

During radiotherapy, the occurrence and extent of acute radiation toxicity (such as hematological toxicity, liver and kidney function, gastrointestinal reaction, local mucosal response after intracavitary radiotherapy, etc.) will be regularly assessed, and the interruption of radiotherapy will be avoided as far as possible. If the patient has serious rectal, bladder reactions and other serious radiation side effects, and the treatment effect is poor, radiotherapy will be suspended. The causes and days of radiotherapy interruption should be recorded in detail.

### Chemotherapy

Weekly cisplatin at a dose of 30 mg/m^2^ was administered for five courses during the radiotherapy period. The first course of cisplatin was administered on day 1 of radiotherapy. Cisplatin could be given on the same day as HDR-ICBT and external beam radiation therapy. Two to four cycles of sequential TP (Taxol 135 mg/m^2^, D1 and DDP 75 mg/m^2^, D1–3) would be performed according to complete relief (CR).

### Dose adjustment of paclitaxel

Dose adjustment of paclitaxel should be performed when neutropenia and/or its complications are observed (Tables 3 and 4), or with nausea and/or vomiting. Starting from the first chemotherapy cycle, 5-HT3 antagonists should be given to prevent vomiting. In addition, corticosteroids for 3 days to prevent fluid retention can further reduce the incidence and severity of vomiting. Patients who are still experiencing nausea and vomiting after taking these measures may use other appropriate antiemetic regimens.

**Table 3:** Febrile neutropenia or infection

Adverse event	Measures
● Febrile neutropenia ● Infection	1. For first-time febrile neutropenia or infection, G-CSF should be added to all subsequent cycles 2. If the attack is repeated, the patient will maintain ciprofloxacin and G-CSF treatment; in addition, the paclitaxel dose will decrease from 75 mg/m^2^ to 60 mg/m^2^ in the follow-up cycle

**Table 4:** Neutrophil count at 21 days

Neutrophil count (× 10^9^/L)	Measures
≥ 1.5	On time
< 1.5	1. Delay for 1 week and repeat the blood cell count at 28 days 2. If absolute neutrophil count (ANC) is more than 1.5 × 10^9^/L, continue to use full dose chemotherapy without G-CSF 3. If ANC < 1.5 × 10^9^/L, consider adding G-CSF for 7 days on day 28 ● Blood count on day 35 ● If ANC is more than 1.5 × 10^9^/L, continue to use full dose chemotherapy 4. In follow-up cycles consider using G-CSF 5. If the patient fails to recover at 35 days (ANC < 1.5 × 10^9^/L), terminate chemotherapy

## Abnormality of bilirubin and impaired liver function

When the level of bilirubin is abnormal, the next chemotherapy cycle will be delayed for 2 weeks. If it is not recovered, the patient will be terminated to study chemotherapy. If there is an abnormal AST and/or ALT and/or ALP level when there is no disease progression, the dose will be adjusted according to the protocol in Table 5.

**Table 5:** Liver function

AST/ALT	Alkaline phosphatase	Dose adjustment
< 1.5 × upper normal limit (UNL)	< 5 × UNL	No change
> 1.5 to < 2.5 × UNL	< 2.5 × UNL	No change
> 2.5 to < 5 × UNL	< 2.5 × UNL	Taxol reduction from 135 to 120 mg/m^2^
> 1.5 to < 5 × UNL	> 2.5 to < 5 × UNL	Taxol reduction from 135 to 120 mg/m^2^
> 5 × UNL	> 5 × UNL	Drug delayed for a maximum of 2 weeks; if taxol reduction from 135 to 120 mg/m^2^ is not achieved, treat patient with chemotherapy

If the dose is down due to impaired liver function, no further reduction is recommended if these liver function indicators do not worsen. If the index is deteriorated after the first dose reduction, the chemotherapy regimen will be terminated.

### Dose adjustment of cisplatin in combined chemotherapy

Peripheral sensory and motor neuropathy: Neurological examinations will be carried out before the admission test, every two cycles and patients terminating chemotherapy. When patients present with symptoms and signs of neuropathy, the examination will be more frequent. The corresponding dose adjustments will be as follows: Grade 0 and grade 1, no change of dosage; grade 2, reduction of cisplatin to 60 mg/m^2^; grade 3 or more, termination of chemotherapy.

Ototoxicity: Patients with grade 3 or 4 ototoxicity will be terminated from chemotherapy.

Nephrotoxicity: Creatinine clearance rate will be determined before the start of each chemotherapy cycle when serum creatinine is higher than normal. Follow-up doses will be lowered according to the following requirements: if the creatinine clearance rate (CC) = 60 mL/min, the total dose CC will be double checked at each cycle; if the CC is between 50 and 59 mL/min, the subsequent cycle dose of cisplatin will be adjusted to 60 mg/m^2^; if CC < 50 mL/min, cisplatin will be discontinued in the subsequent cycle.

Thrombocytopenic purpura: if there is < 25 × 10^9^/L, the dose of cisplatin will be reduced from 75 to 60 mg/m^2^.

### Dosage adjustment of platinum class in single drug regimen

According to the classification of adverse reactions of cisplatin in the dual drug regimen, the dosage of corresponding cisplatin will be decreased from 30 to 25 mg/m^2^/w.

### Dose adjustment of synchronous chemotherapy

Blood routine examination and blood biochemistry will be checked before each chemotherapy. Adequate chemotherapy will be given strictly according to the treatment plan, and dose adjustment is not permitted under any circumstances. If neutrophil < 2.0 × 10^9^/L or platelet < 100.0 × 10^9^/L, chemotherapy time could be delayed. If inosine clearance rate is less than 50 mL/min, chemotherapy will be suspended.

### Overdose

Cisplatin overdose: if the drug dose exceeds 120 mg/m^2^, its toxicity would increase, especially nephrotoxicity and hematological toxicity. Paclitaxel overdose: The most important predictable overdose complications include myelosuppression, peripheral neurotoxicity, and mucositis.

### Storage conditions, labeling, accountability, and chemotherapy preparation

#### Storage

Drugs are stored in a dedicated storage refrigerator and saved under preservation conditions. Paclitaxel is preserved in light and sealed at 2–8 °C. Cisplatin is stored in the dark and sealed at room temperature.

#### Labeling

Drug name, quantity, receiving time; formulation and dosage, batch number and expiration date; preservation conditions and precautions; newly received and returned sponsor’s drug count will all be adequately labelled.

#### Accountability

The use of drugs is the sole responsibility of the investigator. Drugs for clinical trials are prohibited from being sold and it is strictly prohibited to collect fees for the subjects who use the trial medications. The investigator will ensure that all experimental drugs are used only for the subject in the clinical trial, and that the dose and usage strictly follow the protocol. The investigator will not transfer the drugs to any non-clinical test participants or use them for other purposes**.** According to the test plan, the ‘clinical test drug subject release/recovery registration form’ will be filled out.

### Chemotherapy preparation

The test drugs will be inspected, including properties, packaging, efficacy, storage temperature, etc.

Prior to dosing, the researcher will recheck the identity of the subject based on the identification wristband and confirm that she has completed all procedures prior to administration according to the protocol and that she meets the inclusion criteria.

Standard blood tests, radiologic tests, liver and kidney function tests, and ECG test will be performed. Further, the researcher will determine whether the test and calibration of the infusion pump is within the validity period and whether the instrument is operating normally.

The patient will be informed about the side effects of medication.

### Reporting of safety events

The research doctor will report to the project leader, food and drug supervision and management department, sponsor, ethics committee, and drug clinical trial office within 24 h of any safety events. Research doctors should immediately take appropriate treatment for patients with adverse events. Research doctors will make serious adverse event records, including description of adverse events, time of occurrence, time of termination, degree and frequency of attacks, and treatment given.

### Follow-up

The response will be assessed by MRI T2-weighted images 2 months after the completion of treatment according to the RECIST criteria. Patients will be followed every 2 months for the first 2 years. Follow-up includes a pelvic examination with PAP smear and monitoring of tumor markers if initially elevated. CT scans of the abdomen and pelvis and chest x-ray (or CT scan) performed annually. Pelvic disease progression is defined as follows: pelvic recurrence after the assessment of complete remission, pelvic disease progression with a > 20% increase in the size of target lesions assessed by MRI T2WI, or the initiation of salvage treatment for pelvic disease.

### Insurance/indemnity

Physicians will do their best to prevent and treat the adverse reactions that may be caused by this study. If an adverse event occurs in clinical trials, the Medical Expert Committee will identify whether it is related to Radiotherapy or basic treatment drugs. The sponsor will provide the cost and economic compensation in accordance with the provisions of the “Quality Management Practice for Drug Clinical Trials” in China.

### Statistical considerations

In our study, PFS was conducted for the primary outcome. We need to include 220 participants in our analyses (110 per group). Given the 30% loss to follow-up, we need to recruit 300 women for our research.

All patients will be followed up every 2 months for 2 years and every 3 or 6 months thereafter. All acute toxicity and late toxicity will be graded according to the toxicity criteria of the RTOG and the EORTC [24]. The treatment effect will be evaluated in terms of local control, PFS, and overall survival. Local control is defined as no evidence of tumor regrowth or recurrence in the treatment volume according to physical examination, CT, MRI, PET, and/or biopsy. Local control, PFS and overall survival rates are calculated by the Kaplan–Meier method, performed with SPSS 20.0. All the data will be collected in a paper case report form.

### Protocol deviations

At the project evaluation stage prior to the start of the trial, the clinical trial investigator carefully read the program and fully discussed the feasibility with the sponsor.

The investigator will explain the importance of the compliance program to the subject when informed consent is obtained.

After receiving a ‘violation of the scheme report’, the research institution will analyze the causes. If it is due to negligence of the organization or a lack of understanding of the program, the researcher will be supervised to improve and avoid similar situations from happening again. If it is serious or persists, the institution will suspend the investigator’s participation in clinical trials. If it is due to poor conditions, the research institution will improve conditions in a timely manner. The research institution periodically classifies and summarizes all protocol deviations in order to take appropriate measures to improve the system’s quality management of clinical trials.

## Discussion

RapidArc is a novel form of radiotherapy delivery that allows the radiation dose to be delivered in a single 360 gantry rotation. It could be considered as a treatment for a select group of cervical cancer patients, and aims to improve the degree of target coverage, improve OARs, spare healthy tissue compared to other IMRT solutions, and significantly reduce the treatment time.

A series of studies showed that RapidArc has an obvious advantage in the dose distribution and organ protection. In one study performed by Cozzi et al. [5], RapidArc showed a slightly improved target coverage compared to IMRT in terms of D2% and D5%-D95%. The results from the dose–volume histogram analysis showed a systematic and highly statistically significant reduction in OAR involvement with RapidArc compared to IMRT. Palma et al. [29] investigated the RapidArc progenitor on the prostate, showing that variable dose rate volumetric arc modulation is beneficial compared to IMRT or a constant dose rate. Zhai et al. [4] investigated RapidArc on whole pelvic lymph nodes in cervical cancer; RapidArc plans resulted in fewer monitor units than the corresponding f-IMRT plans.

Anatomical images lack the sensitivity to define tumor extent and the capacity to evaluate the biology of the tumor and normal tissue. Several studies have indicated that FDG-PET/CT increases the agreement between biopsies and delineated tumor volume and has the potential to impact the course of treatment [12, 13]. In external beam radiotherapy and brachytherapy, accurately defining disease areas is critical to avoiding normal tissue. Based on FDG-PET, higher doses to the FDG-avid tumor volume can be safely delivered, while relatively low doses to the bladder and rectum can be expected [12, 14]. In Kidd et al.’s study [18], 452 patients with newly diagnosed cervical cancer who were treated with FDG-PET/CT-guided IMRT showed improved survival and less treatment-related toxicity. Lin et al. [30] conducted a dosimetric study comparing intracavitary brachytherapy using a standard plan with a PET-defined tumor volume in 11 patients undergoing intracavitary treatments. They concluded that FDG-PET-based treatment planning improved tumor dose coverage without significantly increasing doses to the bladder and rectum.

Increasing tumor cell treatment sensitivity could be helpful to improve local control and survival. The Comet assay is attractive as a potential clinical test of tumor radiosensitivity, as it requires fewer cells and generates results rapidly compared with standard clonogenic and other electrophoresis assays [21–23, 31]. In a series of seven cervical tumor cell lines, Marples et al. [10] indicated that the Comet assay can be used to predict the radiosensitivity of cervical tumor cell lines by assessing the ratio of initial and residual DNA double-strand breaks. Bowman et al. [9] demonstrated that mechanisms governing treatment-induced DNA damage are both central to and predictive of sensitivity to bladder cancer cell treatment and exemplify a link between DNA damage resistance and both treatment response and tumor aggression.

We assumed that RapidArc-IMRT could provide a better radiation biological effect and higher treatment accuracy, and that FDG-PET/CT and the Comet assay could provide more precise tumor and local radiation sensitivity information. A more precise target volume will be established and higher doses of irradiation could be delivered to the tumor region.

### Trial status

This report describes the protocol from May 1, 2017, and adheres to the SPIRIT reporting guidelines with the attached checklist and figure (Fig. 1 and Fig. 2). The first study participant was randomized on July 2016. As of April 1, 2018, 111 of 300 study participants have been enrolled. The targeted end date for recruitment is April 2019.

## Abbreviations

CC: creatinine clearance rate
CCRT: Cisplatin-based chemoradiation
CT: computed tomography
CTV: clinical target volume
EORTC: European Organization for Research and Treatment of Cancer
f-IMRT: forward-planned intensity modulated radiation therapy
FDG: F-18 fluorodeoxyglucose
GTV: gross target volume
HDR-ICBT: High-dose rate intracavitary brachytherapy
IMRT: intensity modulated radiation therapy
LACC: locally advanced cervical cancer
OARs: organs at risk
PET: positron emission tomography
PFS: progression-free survival
PTV: plan target volume
RTOG: Radiation Therapy Oncology Group
SUVmax: maximum standard uptake value
